# The Influence of SV40 polyA on Gene Expression of Baculovirus Expression Vector Systems

**DOI:** 10.1371/journal.pone.0145019

**Published:** 2015-12-14

**Authors:** Tamer Z. Salem, Craig P. Seaborn, Colin M. Turney, Jianli Xue, Hui Shang, Xiao-Wen Cheng

**Affiliations:** 1 Department of Microbiology, 32 Pearson Hall, Miami University, Oxford, Ohio, United States of America; 2 Biomedical Sciences, University of Science and Technology at Zewail City, Giza, Egypt; 3 Department of Microbial Molecular Biology, AGERI, ARC, Giza, Egypt; Wuhan Bioengineering Institute, CHINA

## Abstract

The simian virus 40 polyadenylation signal (SV40 polyA) has been routinely inserted downstream of the polyhedrin promoter in many baculovirus expression vector systems (BEVS). In the baculovirus prototype *Autographa californica* multiple nucleopolyhedrovirus (AcMNPV), the polyhedrin promoter (very late promoter) transcribes its gene by a viral RNA polymerase therefore there is no supporting evidence that SV40 polyA is required for the proper gene expression under the polyhedrin promoter. Moreover, the effect of the SV40 polyA sequence on the polyhedrin promoter activity has not been tested either at its natural polyhedrin locus or in other loci in the viral genome. In order to test the significance of adding the SV40 polyA sequence on gene expression, the expression of the enhanced green fluorescent protein (egfp) was evaluated with and without the presence of SV40 polyA under the control of the polyhedrin promoter at different genomic loci (polyherin, ecdysteroid UDP-glucosyltransferase (egt), and gp37). In this study, spectrofluorometry and western blot showed reduction of EGFP protein for all recombinant viruses with SV40 polyA, whereas qPCR showed an increase in the *egfp* mRNA levels. Therefore, we conclude that SV40 polyA increases mRNA levels but decreases protein production in the BEVS when the polyhedrin promoter is used at different loci. This work suggests that SV40 polyA in BEVSs should be replaced by an AcMNPV late gene polyA for optimal protein production or left untouched for optimal RNA production (RNA interference applications).

## Introduction

The insect specific baculoviruses in the family of *Baculoviridae* have been widely used for high yield expression of heterologous proteins in insect cells for research and pharmaceutical applications [1,2,3,4]. This is attributed to the fact that the large circular dsDNA genome of baculovirus (88–180 kb) has genes that are dispensable and can be replaced with foreign genes for expression purposes [5,6]. For example, in the genome of the most extensively studied baculovirus, *Autographa californica* multiple nucleopolyhedrovirus (AcMNPV), the highly expressed *polyhedrin* (*polh*) and *p10* genes are not essential for AcMNPV replication in cell culture [7,8]. This discovery leads to the development of the baculovirus expression vector system (BEVS) [7]. The BEVS has at least three major attractive advantages over other systems for gene expression. First, the strong promoters such as those of *polh* and *p10* allow abundant expression of foreign genes. Second, they support the proper production of the mammalian proteins in insect cell culture or in live insects [9]. Third, the mechanisms for post-translational modification of proteins in insect systems are similar to those in mammalian systems [1,10].

Two different groups of genes are classified depending on whether they are transcribed prior to or posterior to viral DNA replications. Early genes are transcribed by the host RNA polymerase (POL) II without the need of viral DNA replication. However, the late genes that are transcribed by the viral RNA POL, driven by an early promoter, are transcribed posterior to viral replication [11]. The *polh* promoter is a strong promoter that drives the expression of a late gene (polyhedrin gene) and has been widely used for protein production in the vast majority of the BEVSs [1,2].

To further enhance protein production in the BEVS, a 128 bp simian virus 40 (SV40) polyadenylation signal sequence or SV40 polyA has been routinely added to some of the *polh* promoter-based transfer vectors such as the popular Bac-to-Bac^®^ pFastBac^™^ vectors and Gateway^®^-adapted destination vectors (Invitrogen). The SV40 polyA signal is recognized and used by the host RNA POL II complex to process precursor mRNA and increase the stability of the mature mRNA as well as enhance the efficiency of mRNA translation in eukaryotic cells. Therefore, its insertion in the BEVS is intended to provide efficient mRNA processing and polyadenylation and to boost protein expression levels in insect cells. Although critics suggest that additional polyadenylation signals should not be added when foreign genes are to be expressed in the BEVS, the significance of adding polyadenylation signals has not been fully addressed [12]. Early work suggests that the insertion of SV40 polyA at the *p10* locus in other BEVSs reduces mRNA production and thus reduces protein synthesis [13]. However, the role of SV40 polyA in the *polh* promoter-based vectors has not been systematically investigated. Therefore, we designed different experiments to investigate the influence of using SV40 polyA on enhanced green fluorescent protein (EGFP) expression, which is driven by the polyhedrin promoter in three different loci on the AcMNPV genome. Recording the influence of using SV40 polyA on foreign genes driven by late promoters in BEVS is very important to the baculovirus-based applications such as vaccines, pharmaceutical products and RNA interference.

## Materials and Methods

### Cell line and viruses

The insect cell line IPLB-SF21AE (Sf21) used throughout this investigation was maintained at 27°C in the TNM-FH medium supplemented with 10% fetal bovine serum. AcMNPV (E2 strain) was used to test the significance of SV40 polyA in the BEVS. The Bac-to-Bac^®^ system was obtained from Invitrogen.

### Recombinant virus construction

Three viral loci were used to test the roles of SV40 polyA in gene expression levels in the BEVS. Transfer vectors were constructed to generate three recombinant viruses that express *egfp* in three independent loci (*polh*, *egt* and *gp37*).

**Fig 1:**
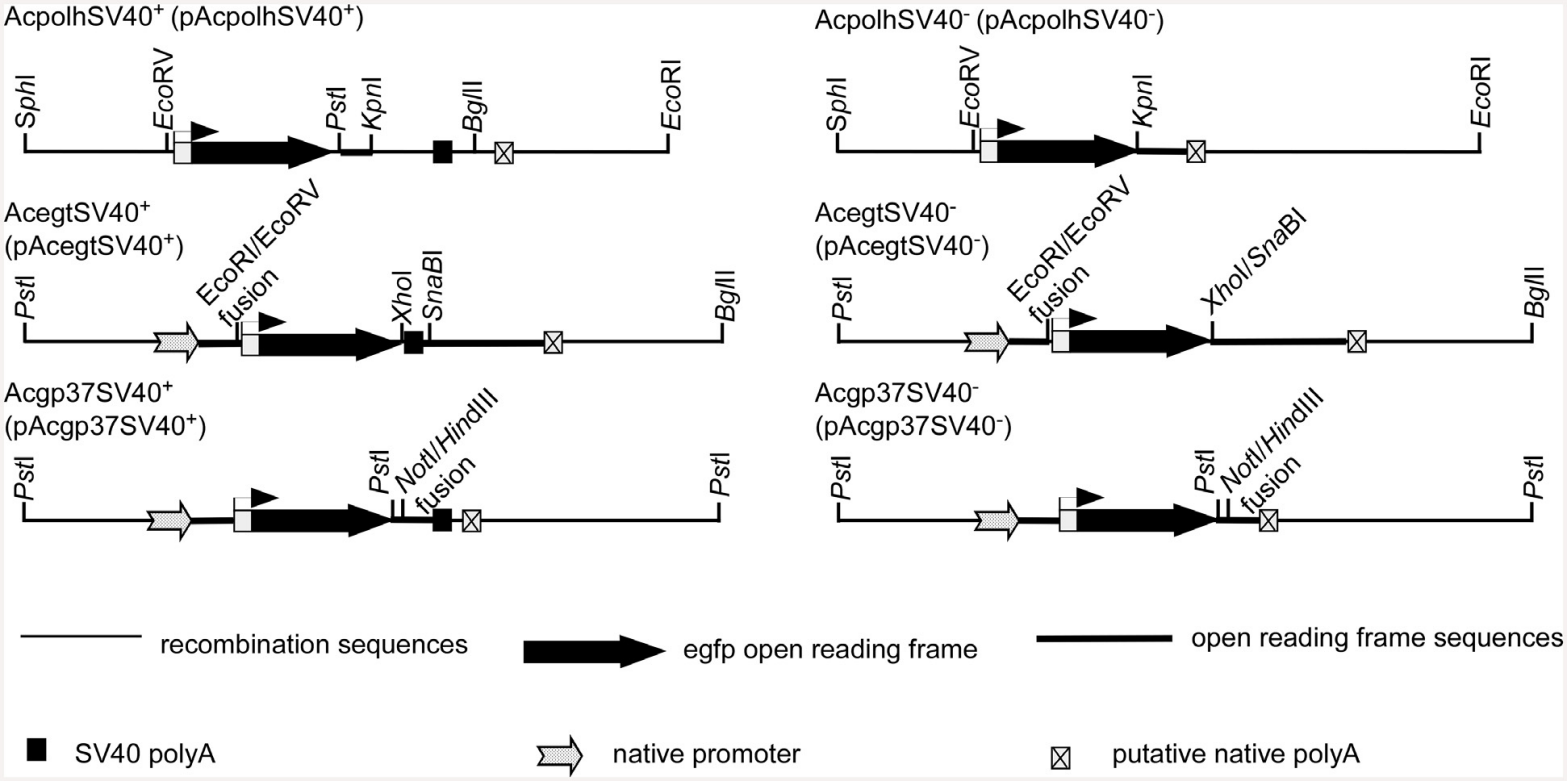
Schematic diagram of viral constructs with and without SV40 polyA signals for egfp gene expression under the AcMNPV *polh* promoter control at the *polh*, *egt* and *gp37* loci of AcMNPV genome to test the functions of SV40 polyA in the baculovirus expression vector system. The putative polyA signal in Acgp37SV40+ and Acgp37SV40- is a prediction but not verified since no 3’ RACE products were obtained.

At the *polh* gene locus, for the SV40^-^ construct, a 5.2 kbp *Eco*RI/*Sph*I fragment of AcMNPV containing *polh* was cloned between the *Eco*RI and *Sph*I sites of the pUC18 plasmid. The resultant 7.9 kbp cloned (pUCpolh) DNA was cleaved with *Eco*RV/*Kpn*I to delete the *polh* promoter and a major portion of the *polh* coding sequence (631 bp). A clone, pBlueGFP containing the *egfp* gene, was digested with *Eco*RV/*Kpn*I to retrieve the *polh* promoter and the *egfp* gene [14]. The 0.9 kbp *Eco*RV/*Kpn*I *egfp* fragment was ligated to the 7 kbp *Eco*RV/*Kpn*I fragment from pUCpolh to generate pAcpolhSV40^-^ (Fig 1).

For the SV40^+^ construct, the fragment (400 bp) containing the SV40 polyA signal was retrieved from pBlueBac4.5 (Invitrogen) and inserted downstream of the *egfp* gene in the transfer vector pAcpolhSV40^-^ to produce a clone carrying the *polh* promoter upstream of the *egfp* gene with downstream SV40 polyA (pAcpolhSV40^+^) (Fig 1).

At the *egt* gene locus, for the SV40^+^ construct, a 3.8 kbp *Pst*I/*Bgl*II fragment of AcMNPV was cloned between the *Pst*I and *Bam*HI sites of pUC18E in which the *Eco*RI site was previously eliminated by Klenow enzyme treatment (pUC18E-egt, 6.5 kbp). The plasmid pUC18E-egt was digested with *Eco*RI/*Sna*bI and followed by Klenow enzyme treatment for blunt end production. The *egfp* gene with the *polh* promoter at the upstream and SV40 polyA at the downstream was retrieved from pBlueGFP [14] by digestion with *Eco*RV/*Sna*bI (blunt ends) and was ligated into the pUC18E-egt to produce transfer vector pAcegt SV40^+^ (Fig 1).

For the SV40^-^ construct, the SV40 polyA signal in pAcegtSV40^+^ was deleted by double-digestion with *Xho*I and *Sna*BI followed by Klenow enzyme treatment. Thus treated plasmid was ligated by T4 DNA ligase to produce a transfer vector called pAcegtSV40^-^ (Fig 1).

At the *gp37* gene locus, for the SV40^-^ construct, the transfer vector pAcGFP or in this report known as pAcgp37SV40^-^ was used [14]. For the SV40^+^ construct, the SV40 polyA signal was inserted downstream of the plasmid pAcgp37SV40^-^ [14] to generate transfer vector pAcgp37SV40^+^ that contained the SV40 polyA sequence (Fig 1).

All transfer vectors were confirmed by restriction endonuclease and DNA sequence analyses. For each viral construct, the transfer vector and AcMNPV viral DNA were used to co-transfect Sf21 cells using lipofectin (Invitrogen) [12]. Recombinant viruses were isolated by plaque assay and authenticated by PCR [12,14].

To support EGFP expression level differences, the AcMNPV polyhedrin gene open reading frame was amplified by PCR using two primers (Ac-Polh-F-EcoRI and Ac-Pol-R-XbaI, S1 Table) using AcMNPV E2 strain genomic DNA as the template and *pfu* (ClonTech). The PCR product was cloned to the pFastBac I vector that has an SV40 polyA signal to generate pFB-polh-SV40UTR. To insert the AcMNPV polyhedrin UTR, a PCR product was amplified using primer Ac-Polh-F-EcoRI and primer Ac-polhUTR-R-XhoI (S1 Table) that is located 1,209 bp downstream of the polyhedrin translation start codon ATG and AcMNPV E2 strain genomic DNA as the template and *pfu*. The PCR product was cloned into pFastBac I to generate pFB-polh-polhUTR. All the clones were sequenced for confirmation. The two transfer vectors pFB-polh-SV40UTR and pFB-polh-polhUTR were used to transform DH10Bac cells to produce recombinant viruses AcBacPolh-SV40UTR and AcBacPolh-polhUTR in Sf21 cells, respectively, following recommended methods provided by Invitrogen.

### Assay for protein expression

Quantitative analyses of EGFP production driven by the *polh* promoter and in the presence or absence of SV40 polyA at the *polh*, *egt* and *gp37* loci were carried out as follows. Sf21 cells were infected in triplicates by different recombinant viruses and wt AcMNPV at an m.o.i. of 1 and10. MOI was calculated based on the plaque forming units (p.f.u.) per cell in 25 cm^2^ TC flasks containing 3×10^6^ cells/flask [12]. The infected cells were incubated for 72 h (1 m.o.i.) and 48 h (10 m.o.i.) at 27°C. After incubation, the infected cells were dislodged from the TC flasks. One ml of suspended cells was withdrawn for SDS-PAGE and western blot analysis. Two ml of cells were taken out for EGFP fluorescence measurement and the remaining 2 ml were used for total RNA isolation.

To measure the EGFP yield, infected Sf21 cells were centrifuged at 400 g for 5 min and the pellets were lysed in 1 ml of 0.1% SDS. The lysate containing EGFP was measured for fluorescence using a Shimadzu RF-5301PC spectrofluorometer (Ex 488 nm, Em 507 nm). The emission values were used to quantitatively compare the expression levels of EGFP between different pairs of recombinant viruses. The results were analyzed by the Student’s T-test in Excel (Microsoft).

To ensure that the EGFP measurement was in the linear range, a flask of Sf21 cells were infected with AcegfpSV40^-^ and the infected cells were harvested as described above. Sf21 cells were serially diluted 2-fold with 0.1% SDS up to 8-fold. The EGFP fluorescence intensity was measured. Correlation of EGFP emission and cell numbers were analyzed by linear regression using the program Excel (Microsoft).

To confirm the EGFP fluorescence measurement, SDS-PAGE and western blot analyses were performed. Infected Sf21 cells were divided equally. One was used for the total protein estimate and the other half was used for western blot analysis. In total protein analysis, the infected cells were pelleted by centrifugation and lysed in 0.1% SDS. A Bradford based Bio-Rad protein assay kit was used to estimate the total proteins in infected cells following the protocol provided by the kit provider (Bio-Rad, Hercules, California). Known amounts of bovine serum albumin (BSA) were used to construct a standard curve. In western blot analysis, equal amounts of total protein (10 μg) from each viral infection were processed for SDS-PAGE in triplicates. One of the gels was stained with Coomassie blue. Proteins in the other two gels were transferred onto two nitrocellulose membranes separately. One membrane was used for EGFP detection with an anti-GFP polyclonal antibody (Affinity BioReagents, Golden, Colorado). The other nitrocellulose membrane was used for the detection of a viral capsid protein VP39 with an anti-VP39 mono-clonal antibody (kindly provided by Dr. L. E.Volkman, University of California, Berkeley) for normalization of sample loading. Horseradish peroxidase conjugated secondary anti-bodies were used to bind to either the EGFP or VP39 on the membranes. An Immun-Blot Assay kit was used for color development following the protocol from the manufacturer (Bio-Rad). The procedure was performed in triplicates from cell infection to the western analyses.

To ascertain that the detection was in the linear range, the EGFP expression from AcegtSV40^-^ infected Sf21 cells were also serially diluted (2-fold in each dilution) for western blotting analysis with the Anti-GFP anti-body. The signals from western blots were quantified by spot densitometry using a computer program AlphaImager 2200 (Alpha Innotech Corporation, California). Differences in GFP antibody signals between each pair of recombinant viruses were analyzed statistically using the Student’s T-test in Excel (Microsoft).

To support EGFP measurements, polyhedrin expression differences between AcBacPolh-SV40UTR and AcBacPolh-polhUTR in Sf21 cells were compared at an m.o.i. of 5 in triplicates. At day 4 post infection, the infected cells were lysed with 0.5% SDS for purification of polyhedra by centrifugation (16,000 g for 1 min). The pelleted polyhedra were washed three times by 0.5% SDS. The amounts of polyhedra production between the two viruses in Sf21 cells were enumerated using a hemocytometer.

### Transcription level analysis

To test the differences in *egfp* transcripts between viruses with and without SV40 polyA, real-time qPCR was used. The other half of cell pellets used to measure protein expression above was used for total RNA extraction [15]. The extracted RNA was first quantified by spectrophotometry. Total RNA (1 μg) was treated with DNase (Promega) to degrade potential DNA contamination following conditions recommended by the enzyme provider. The DNA-free RNAs were then used as templates for cDNA synthesis using two reverse primers, an oligo (dT) 3’ RACE adapter primer (Ambion, Table in S1 Table) and a Sf28-R primer, together in the reaction with a DyNAmo cDNA Synthesis Kit (New England Biolabs, Beverley, USA) [16]. The synthesized cDNA was diluted 4-fold with nuclease-free water for qPCR analysis of *egfp* transcript levels. In qPCR, the primer pairs used for a 153 bp amplicon of *egfp* gene were pGFP-486F and pGFP-639R (S1 Table). An internal system control with the primer pair of SF28S-F and SF28S-R for the Sf21 28S rRNA was used to normalize the reactions. SYBR^®^ Green supermix kits (Bio-Rad) were used in the real-time qPCR [16]. The amplification data was acquired by the Bio-Rad IQ 3.0 system. Effects of SV40 polyA on *egfp* transcript levels were expressed as relative to the 28S rRNA levels [17,18]. Each of the amplifications was run in triplicates (templates from three independent infection) to calculate experimental variance for statistical analysis by the Student’s T-test using Excel (Microsoft).

To support real-time qPCR measurement of EGFP expression, dot-blot RNA analysis was performed. Equal amounts (2 μg) of total RNA from each viral infection were blotted onto a nylon membrane (OSMONICS INC) in triplicates with an S& S Minifold (Schleicher & Schuell) following the provided procedure. A pair of *egfp* primers, GFP-112F and GFP-506R (S1 Table), was used to amplify a 394 bp PCR product using pBlueGFP plasmid [14] as a template and the PCR product was labeled with a biotin labeling kit (NEB). A partial Sf21 cellular *28S* ribosomal RNA gene sequence was cloned (pGEM-sf28S) during a cDNA synthesis experiment and was sequenced (GenBank Accession. No: EU314585). The *28S* gene fragment was amplified using a primer pair of SF28S-F and SF28S-R (S1 Table) by PCR using the plasmid pGEM-sf28S as a template for an expected 157 bp product and labeled with the biotin labeling kit (NEB). One of the dot blot duplicates was hybridized by the *egfp* biotin probe and the other was hybridized by the *28S* rRNA biotin probe in formamide based hybridization buffers [14] at 42°C for 19 h. the blots were processed for color development following the protocol by the kit supplier (NEB).

### Mapping of 3’ends

To map the 3’ ends of the transcripts, the 3’ RACE procedure was used. A cDNA was synthesized by using RNA isolated above and an oligo (dT) 3’ RACE adapter primer (Ambion, S1 Table) according to the instruction of the cDNA synthesis kit (NEB). A forward *egfp* gene primer (GFP-486F, S1 Table) was paired with the 3’ RACE outer reverse primer (Ambion, S1 Table) to amplify the 3’ ends by PCR using synthesized cDNA templates. The amplified products were resolved by agarose gel electrophoresis. The RACE products from the agarose gel were cloned for sequencing. The obtained sequences were compared with the genome sequence of AcMNPV to map the 3’ ends using LASERGENE (DNASTAR). If no RACE products were detected, *egfp* gene specific primers were used to test the presence of *egfp* cDNA. The *egfp* gene specific primers were GFP-112F and GFP-506R (S1 Table) for an expected 394 bp PCR product.

### Analysis of gp37 UTR

To understand regulation of *egfp* expression with the *gp37* UTR, the AATAAA polyadenylation signal was first searched using LASERGENE (DNASTAR). Oligo primers located immediately upstream of the AATAAA were designed and synthesized for PCR analysis (S1 Table). In PCR, each of the four *gp37* UTR reverse primers (S1 Table) was paired with the forward primer GFP-486F (Fig 1, S1 Table) with the cDNA from above as well as with the plasmid pAcgp37SV40^-^ DNA. The amplified products were analyzed by agarose gel electrophoresis.

## Results

### Protein expression yield analysis

**Fig 2:**
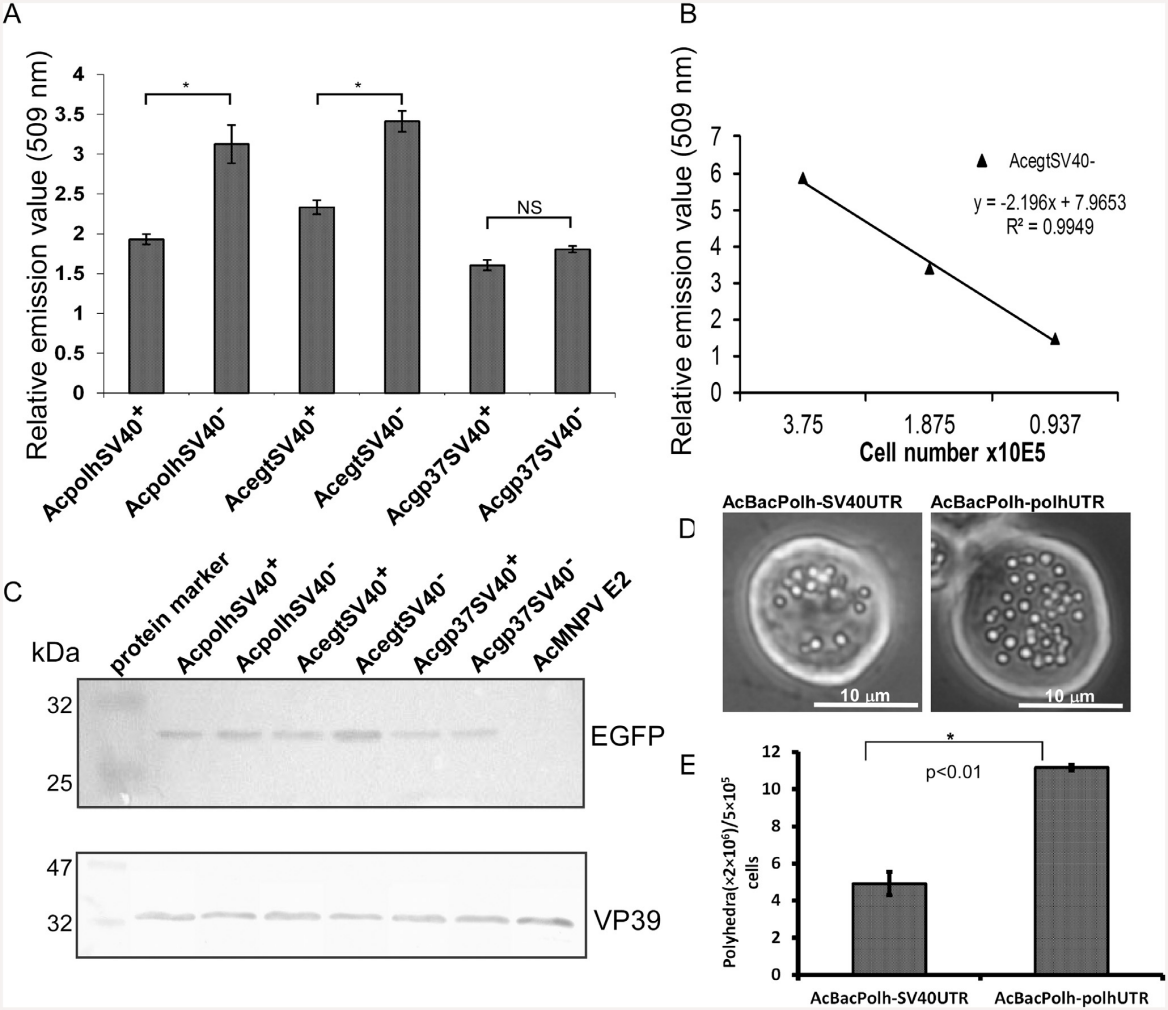
EGFP expression yield comparison of Sf21 cells infected with viral constructs with or without the SV40 polyA signal at the polh, egt and gp37 loci of the AcMNPV genome. **A**. Quantitative analysis of EGFP yields to test the effects of SV40 polyA on EGFP expression in the baculovirus expression vector system. Sf21 cells were infected by different viral constructs at an m. o. i. of 10 p.f.u./cell and the cells were harvested at 48 h post infection for fluorescence emission measurement in three independent cell infections. * indicates significant difference at *P* = 0.05. NS, not significant. Error bars denote SD. **B**. Validation of the fluorescence measurement to ascertain fluorescence values falling in the linear range. EGFP produced by the Sf21 cells infected by AcegtSV40^-^ were diluted and fluorescence emission values were plotted against the number of cells. **C**. Western blotting analysis of EGFP yields by viral constructs with or without the SV40 polyA signal. Equal amounts of total proteins from Sf21 cells infected with viruses were used for SDS-PAGE. The separated proteins from the gel were transferred onto a nitrocellulose membrane and probed with an anti-GFP antibody to detect the amounts of EGFP production from different viral infections. A separate membrane identical to that for the EGFP detection was probed with an anti-VP39 antibody. **D**. Comparison of polyhedra production of Sf21 cells infected with either AcBacPolh-SV40UTR or AcBacPolh-PolhUTR. **E**. Quantitative comparison of polyhedra production of Sf21 cells infected with either AcBacPolh-SV40UTR or AcBacPolh-PolhUTR. * indicates significant difference at *P* = 0.05.

Three pairs of recombinant AcMNPV were constructed to express EGFP driven by the *polh* promoter in the presence and absence of the SV40 polyA signal that inserted in different loci at the AcMNPV genome (*polh*, *ecdysteroid UDP-glucosyltransferase* (*egt*) and *gp37* loci) (Fig 1). Fluorescence spectrophotometry was used to measure the level of EGFP expression. All three recombinant viruses lacking the SV40 polyA signal (AcpolhSV40^-^, AcegtSV40^**-**^, Acgp37SV40^-^) showed higher EGFP yields than the constructs that have the SV40 polyA signal (AcpolhSV40^+^, AcegtSV40^**+**^, Acgp37SV40^+^) when they infected Sf21 cells at a multiplicity of infection (m.o.i.) of 10 (Fig 2A). Viral infection at an m.o.i. of 1 showed similar EGFP yield differences as in the 10 m.o.i. (S1 Fig). There was a significant 30% more EGFP production detected during AcpolhSV40^-^ and AcegtSV40^-^ infection than AcpolhSV40^+^ and AcegtSV40^**+**^, respectively (Fig 2A). At the *gp37* locus, the difference of the *polh* promoter activities between the *gp37* pairs (Acgp37SV40^-^ and Acgp37SV40^+^) was about 10% but not statistically different (Fig 2A). To validate the measurements of EGFP yields using fluorescence spectrophotometry, we determined that all the measurements in Fig 2A were in the linear range detected by fluorescence spectrophotometry (Fig 2B). All these data suggest that the difference in the *polh* promoter-based EGFP expression in the constructs with or without the SV40 polyA signal was not due to experimental errors (Fig 2A) or out of the linear range of the EGFP measurements (Fig 2B). Instead, it suggests that the presence of the SV40 polyA signal downstream of the *polh* promoter-based BEVS reduced EGFP expression yields, which is locus dependent. To further support the conclusion from the EGFP fluorescence measurement, SDS-PAGE and western blot were used to visualize the difference between each pair of the viral constructs.

The differences of the EGFP levels were confirmed by western blot (Fig 2C). No EGFP band was detected from the Sf21 cells infected with the wild type (wt) AcMNPV E2 as a negative control. It was clear that the western results for AcegtSV40^-^ showed the strongest signal in the western blot analysis (Fig 2C), which confirmed the fluorescence spectrophotometry results. The anti-VP39 antibody detecting the AcMNPV viral capsid protein VP39 was used as a loading control (Fig 2C). This result suggests that the difference in EGFP expression was not due to the differences in sample loading but due to the negative effects of the SV40 polyA sequence on *polh* promoter-based *egfp* expression. A similar pattern of EGFP reduction to the spectrofluorometry was obtained by densitometry. We also confirmed that the densitometry values were within the linear range (data not shown). All these data suggest that the SV40 polyA signal in the AcMNPV *polh* promoter-based vectors reduced EGFP expression yields at the *polh* and *egt* loci but not at the *gp37* locus of the AcMNPV genome.

To confirm EGFP data, the polyhedra production (corresponding to polyhedrin gene expression) showed a 50% reduction when SV40 poly A sequence was inserted downstream to the polyhedrin gene (AcBacPolh-SV40UTR) comparing to when polyhedrin 5’UTR was inserted (AcBacPolh-polhUTR) (Fig 2D and 2E).

### Analysis of *polh* promoter-based *egfp* transcription

**Fig 3:**
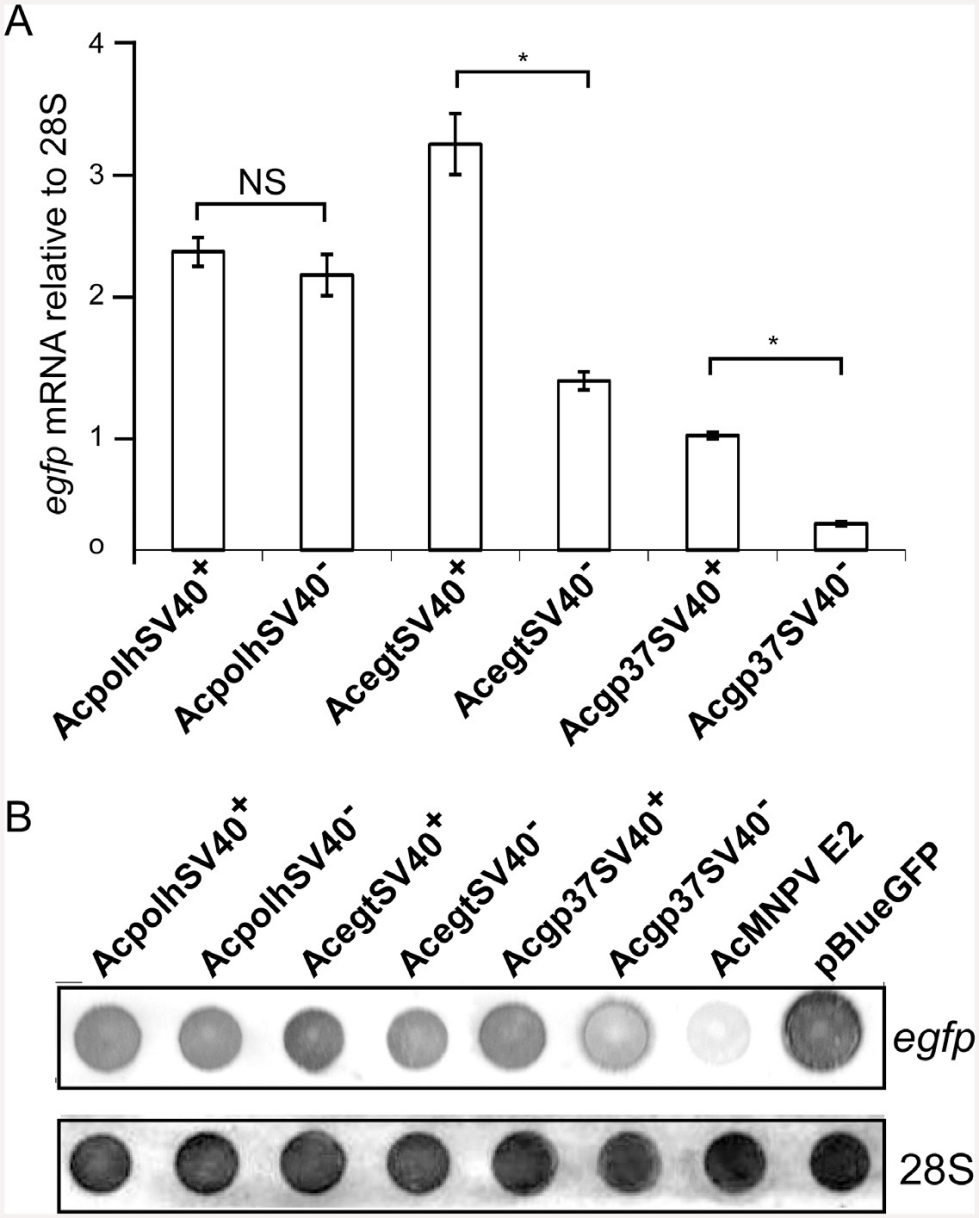
Transcription analysis of *egfp* from different viral infections. Real-time quantitative reverse transcription PCR analysis of *egfp*. Viral constructs with or without the SV40 polyA signal were used to infect Sf21 cells and the infected cells were harvested at 48 h p. i. for total RNA extraction. Equal amounts of RNA from different viral cell infections were used as templates for amplification of a 153 bp amplicon of the *egfp* gene using SYBR green dye RT-PCR kit from Bio-Rad for real-time quantification of the *egfp* transcripts. Sf21 cellular 28S rRNA gene was used as a house keeping gene in the real-time qPCR to normalize the reaction. Error bars represent SD from three independent cell infections. **B**. Dot-blot analysis of *egfp* transcripts. Total RNA (2 μg) from the Sf21 cells infected with AcMNPV with or without the SV40 polyA signal as well as the wt AcMNPV (negative control) and a plasmid pBlueGFP (positive control) were blotted to a nylon membrane and probed with either a *egfp* or 28S rDNA fragment labeled with biotin.

The differences between the *egfp* transcripts from the recombinant viruses with or without the SV40 polyA signal during Sf21 cell infection were initially detected by real-time qPCR [16,17]. The real-time qPCR results showed that the *egfp* transcripts were higher when SV40 polyA signal was used (Fig 3A). However, the increased levels of *egfp* transcripts differed depending on the loci into which the *polh* promoter-based *egfp* expression cassette was inserted. The greatest increase was detected at the *gp37* locus (77%), followed by the *egt* locus (58%) and then the *polh* locus (8%) (Fig 3A). At the *egt* and *gp37* loci, the increased levels of *egfp* transcripts due to the insertion of the SV40 polyA signal was statistically significant compared to the constructs without SV40 polyA, whereas the increase due to the insertion of the SV40 polyA signal at the *polh* locus was not significant compared to that without SV40 polyA (Fig 3A).

The measurements of *egfp* transcription level by real-time qPCR was supported by dot blot RNA analysis that showed differences among *egfp* transcription levels from constructs with and without SV40 polyA. The internal hybridization control using Sf21 *28S* rRNA probe detected almost equal amounts of *28S* rRNA in all the viral infection samples. *28S* rRNA after viral infection was abundantly maintained in the infected cells (Fig 3B). Pair-wise comparison by visual inspection of the hybridization signals showed that higher *egfp* mRNA levels were detected in constructs with SV40 polyA downstream of the e*gfp* gene than these without SV40 polyA at all the three loci (Fig 3B).

Therefore, the SV40 polyA signal at the three loci of the AcMNPV genome examined in this report enhanced *polh* promoter-based *egfp* mRNA levels but reduced EGFP protein levels. These results prompted us to further investigate the 3’ end structure of the *egfp* mRNA initiated from the *polh* promoter at the three loci.

### mRNA 3’ end analysis

**Fig 4:**
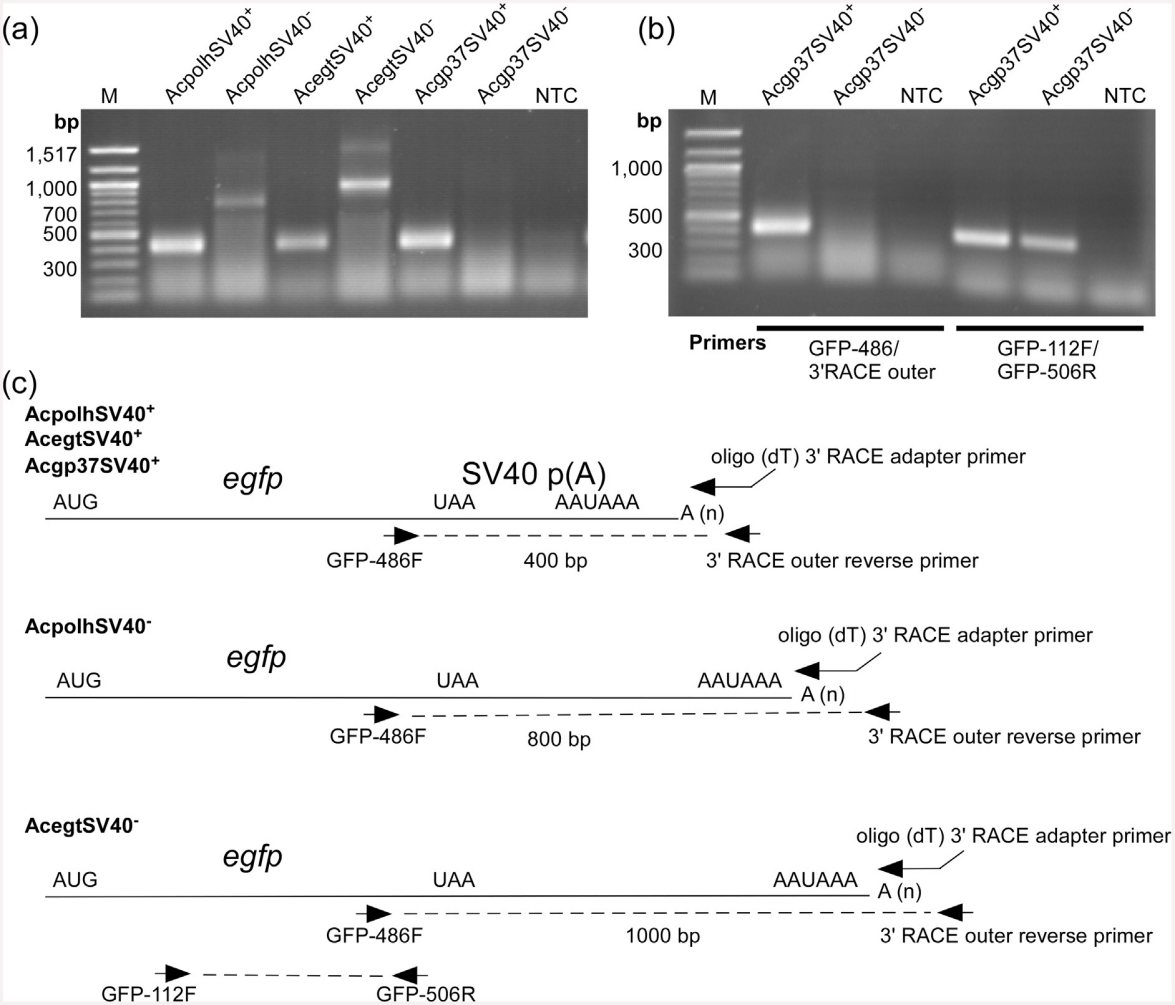
Analysis of untranslated regions of transcripts of viral constructs with or without the SV40 polyA signal in Sf21 cell infection. **A**. 3’ RACE analysis of *egfp* gene transcripts. An oligo (dT) 3’ RACE adapter primer was used to synthesize cDNA from total RNA isolated from the Sf21 cells infected with different viruses at 48 h p. i. A pair of primers (GFP-486F/3’ RACE outer reverse primer) was used to amplify the 3’ ends of the *egfp* transcripts by PCR. The PCR products were separated by agarose gel electrophoresis. **B**. RT-PCR confirmation of the presence of polyadenylated *egfp* mRNA initiated from the *polh* promoter at the *gp37* locus of AcMNPV. NTC, no-template control. M, DNA size marker. **C**. Schematic of *egfp* gene 3’ RACE analysis. Dotted lines indicate 3’ RACE products depicted from **A**.

To determine the 3’ end sequences that might play roles in *egfp* mRNA level regulation at the three loci of the AcMNPV genome, the rapid amplification of cDNA ends (RACE) method was used. For all recombinant viruses with SV40 polyA signal, RACE products of about 400 bp were produced by using the primer pair (GFP-486F/3’outer adapter primer) (Fig 4A and 4C, S1 Table). However, when no SV40 polyA was inserted downstream of the *egfp* gene, the 3’ RACE products were larger than 400 bp for *egfp* at the loci of *polh* and *egt* (Fig 4A and 4C). For AcpolhSV40^-^, a product of about 800 bp was obtained, whereas a product of about 1000 bp was detected from AcegtSV40^-^. No discrete RACE product from Acgp37SV40^-^ was detected by agarose gel electrophoresis (Fig 4A and 4B).

Since PCR product was detected from Acgp37SV40^+^ but not from Acgp37SV40^-^ (Fig 4A and 4B), one of the possibilities could be that there was no cDNA synthesized due to the lack of the poly(A) tail (Fig 4A and 4B, S1 Table). However, using an *egfp* gene specific primer pair (GFP-112F/GFP-506R) (Fig 4B, S1 Table) detected an expected 400 bp product (Fig 4B). These data suggest that the e*gfp* mRNAs produced by Acgp37SV40^-^ were polyadenylated (Fig 4A and 4B). The exact reason for not detecting a 3’ end RACE product with Acgp37SV40^-^ remains unknown, but a plausible explanation could be that there was no efficient polyadenylation signals downstream of the *gp37* 3’ UTR, which led to long *egfp* mRNAs that could not be detected by RACE.

### Mapping of 3’ ends

**Fig 5:**
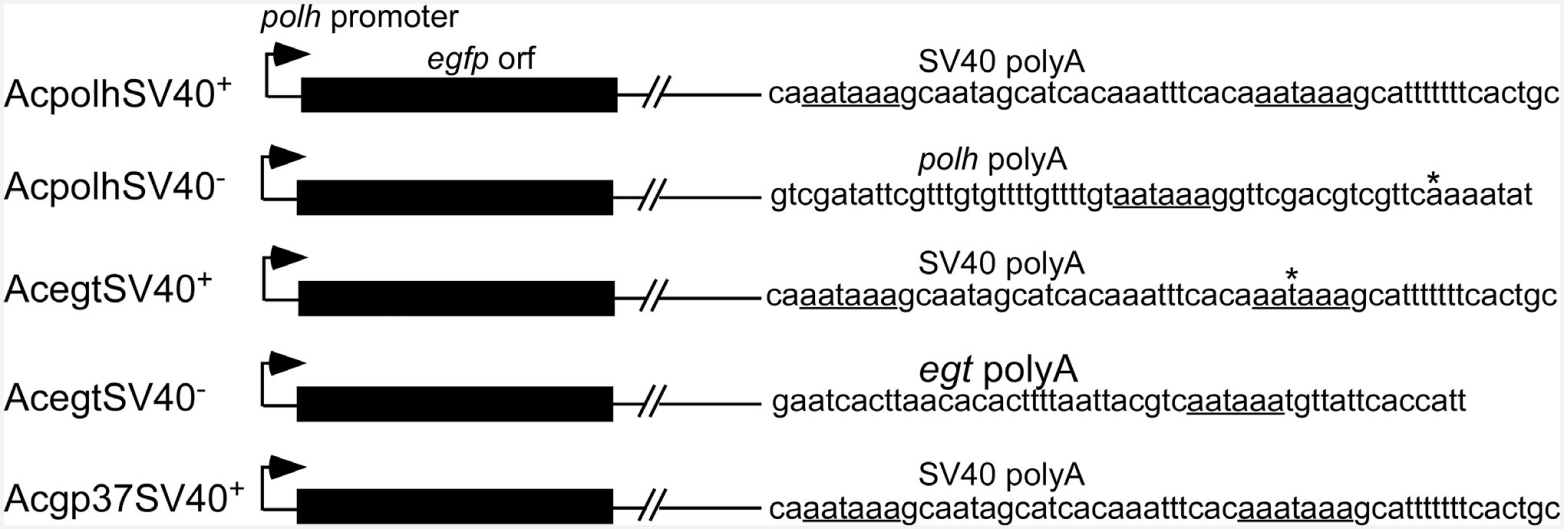
Mapping of the 3’ ends of the *egfp* transcripts from the Sf21 cells infected with different viral constructs with or without the SV40 polyA signal showing the polyA signal (AATAAA) upstream of the 3’ end of transcripts (not drawn to scale). * indicates alternative 3’ ends. // indicates sequences omitted.

To understand why 3’ RACE failed to amplify the 3’ UTR of *gp37*, the RACE products showed in Fig 4a were cloned and sequenced. Sequencing showed that the *egfp* transcripts produced by AcpolhSV40^+^, AcegtSV40^+^ and Acgp37SV40^**+**^ were terminated 16 nt downstream of the AAUAAA motif (Fig 5). When no SV40 polyA was present the *egfp* transcripts were terminated 16–22 nt downstream of the AAUAAA motif at the *polh* locus of AcpolhSV40^-^ and 14 nt at the *egt* locus of AcegtSV40^-^ (Fig 5). Sequencing detected occasional termination within the AAUAAA site for *egfp* transcripts produced by AcegtSV40^+^ (Fig 5). The 3’ end mapping of the SV40 polyA, *polh* and *egt* UTRs suggests that the polyadenylation signal AAUAAA was used by the viral RNA polymerase for the 3’ end processing of *egfp* transcripts that driven by the *polh* promoter. This finding also provided insights in understanding the 3’ end formation of the *gp37* mRNA 3’ UTR.

### Analysis of *gp37* 3’ UTR

**Fig 6:**
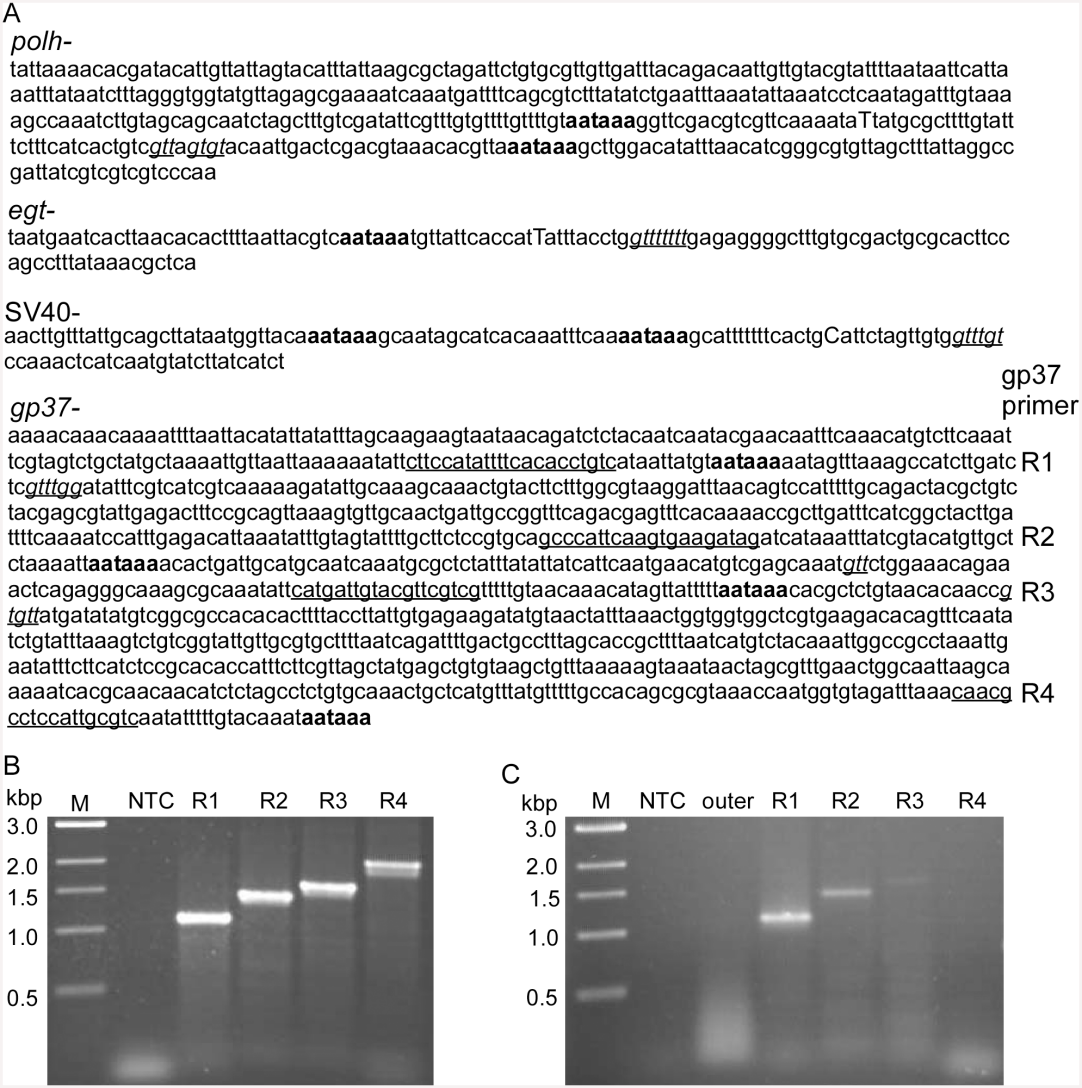
Polyadenylation signal analysis of the *gp37* UTR of AcMNPV. **A**. Comparison of the 3’ UTRs of *polh*, *egt*, *gp37* and SV40. UTR sequences were retrieved from GenBank and searched for the polyadenylation AATAAA and GU-rich motifs. UTR sequences start right after the gene translation stop codon, except SV40 polyA that starts with the 5’ end of the insert. The putative polyadenylation signal AAUAAA or AATAAA is in bold letters. Putative GU-rich motifs are in underlined italic letters. The primary 3’ end nucleotides are in capital letters. Underlines sequences are reverse primers for PCR. **B**. PCR analysis using GFP486F/gp37 reverse primers (R1, R2, R3 and R4) and plasmid DNA template (pAcgp37SV40^-^, Fig 1). NTC, no-template control. M, DNA size marker. **C**. PCR same as B except that cDNA from mRNA isolated from the Sf21 cells infected with Acgp37SV40^-^ (Fig 1). Outer, 3’ RACE outer reverse primer (S1 Table).

A search of the polyadenylation signals in the downstream sequences of *polh*, *egt* and *gp37* as well as the common SV40 polyA revealed the presence of the canonical sequence of AAUAAA and the GU-rich regions needed for 3’ end processing (Fig 6A, Table 1). The putative GU-rich regions were mapped, which were located in the 3’ UTR at 53 nt in AcpolhSV40^-^, 23 nt in AcegtSV40^-^, 25 nt in Acgp37SV40^**-**^ and 28 nt in AcpolhSV40^+^, AcegtSV40^+^ and Acgp37SV40^**+**^ (Fig 6A, Table 1). The majority of the *egfp* transcripts were terminated in the middle between the AAUAAA motif and the GU-rich region (Figs 5 and 6A, Table 1). In the *egfp* 3’UTR of AcpolhSV40^-^, AcpolhSV40^+^, AcegtSV40^+^, and Acgp37SV40^**+**^, two AAUAAA motifs were found. According to the sequence of the 3’ RACE product, the *egfp* transcripts of AcpolhSV40^-^ were terminated close to the first AAUAAA motif, whereas in AcpolhSV40^+^, AcegtSV40^+^ and Acgp37SV40^**+**^ the termination was close to the second AAUAAA motif (Figs 5 and 6A). Interestingly, four AAUAAA motifs are present in the *gp37* 3’UTR between nt 941 and 1,333 from the stop codon (Fig 6A). Using the plasmid DNA (pAcgp37SV40^-^) that includes the1.7 kbp *gp37* 3’ UTR as a template for a PCR reaction as a positive control, four products were produced when the forward primer GFP-486F was used with either of the four *gp37* reverse primers (gp37R1, gp37R2, gp37R3 and gp37R4) (Figs 1 and 6B, S1 Table). When cDNA synthesized from mRNA isolated from the Sf21 cells infected with Acgp37SV40^-^ was used as a template in a similar PCR, only the first three PCR products were detected and their intensity decreased as the length of products increased (Fig 6C). These data suggest that the motifs including the AAUAAA and the downstream GU-rich regions for the 3’ processing of the *egfp* mRNAs produced by Acgp37SV40^**-**^ were not as efficient as the rest of the recombinant viruses (Figs 4 and 6B).

**Table 1:** Comparison of polyadenylation signals of AcMNPV late gene 3’ UTRs with SV40 polyA.

Viral genes	polyA	3' end to polyA (nt)	GU rich region	GU to polyA (nt)
*polh*	AAUAAAgg	22	GUUAGUGU	53
*egt*	AAUAAAtg	14	GUUUUUUU	23
SV40	AAUAAAgc	16	GUUUGU	28
gp37R1	AAUAAAaa	undefined	GUUUGG	25
gp37R2	AAUAAAac	undefined	GUU	66
gp37R3	AAUAAAca	undefined	GUGUU	19

To understand why the first AAUAAA/GU-rich region was not able to efficiently process the *egfp* transcripts at the *gp37* locus, the motifs that are involved in the 3’ end processing were compared between *polh*, *egt*, *gp37* and SV40 polyA (Table 1). The first GU-rich region after the polyadenylation site in the *gp37* UTR showed identical sequence to that of SV40 polyA. This suggests that the first GU-rich region of the *gp37* UTRs might not be responsible for the weak 3’ end processing (Table 1). It is noteworthy to mention that all the AAUAAA of *polh*, *egt* and SV40 polyA are followed by a dinucleotide with at least a guanosine (G), which is not present in the dinucleotide in all three AAUAAA motifs in the *gp37* locus. These data may suggest that additional sequences other than the canonical AAUAAA are needed for the efficient processing of the 3’ ends of the *polh* promoter-controlled transcripts of AcMNPV during Sf21 infection.

## Discussion

Since AcMNPV is the most widely used vector for eukaryotic protein production, the 128 bp SV40 polyA signal has been added to some commercial baculovirus expression vectors including the popular Bac-to-Bac^®^ system (Invitrogen). The impetus to include the SV40 polyA signal into the BEVS is believed to increase protein expression yields. We provided in this report the evidence that the inclusion of the SV40 polyA signal in the BEVS is not only redundant but also reduces protein expression yields. To the best of our knowledge, the most intriguing discovery from this report is that the SV40 polyA signal increased the *polh* promoter-based *egfp* transcription levels but reduced the *egfp* protein accumulation levels of AcMNPV during Sf21 cell infection. The evidence obtained from this work also supports the earlier suggestion that additional polyadenylation signal sequences should not be added in the BEVS to improve protein production yields [12].

In eukaryotic cells, such as Sf21 cells used in this report, the host RNA POL II processes transcripts by recruiting cellular RNA processing factors such as the cleavage and polyadenylation specificity factor (CPSF) and cleavage stimulation factor (CstF) to its carboxyl-terminal domain. CPSF and CstF then recognize a canonical sequence of AAUAAA and a GU- rich sequence downstream of the AAUAAA motif of the transcripts, respectively [19,20]. Other factors poorly understood are then recruited to cleave the sequence between the AAUAAA and the GU-rich region. Subsequently, the poly(A) polymerase adds a stretch of As to form the poly(A) tail and thus stabilize the mRNA in order to enhance protein synthesis [21]. SV40 polyA contains the AAUAAA signal for host CPSF binding [22]. Despite of the discovery that the late genes of AcMNPV are transcribed by the viral RNA POL, the mechanism behind how the AcMNPV RNA POL processes late gene transcripts is not fully understood [11,23]. The fact that the late genes of AcMNPV use the viral RNA polymerase for transcription raises a question about the benefit of the common practice of adding the SV40 polyA signal to improve protein production. However, an empirical evidence of the significance of adding SV40 polyA for baculovirus late gene transcript processing was lacking [12].

Transcription is often an intermediate step in gene expression and the production of functional proteins is the ultimate goal of gene expression using the BEVS. The differences in *egfp* mRNA translation in the Sf21 cells infected with the different recombinant viruses may arise from the 3’ UTRs since they had the same 5’ UTR. Transcripts with poly (A) tails are important for mRNA transportation from the nucleus to the cytoplasm, stability of mRNA and efficient translation [24,25]. Therefore, the length of poly (A) tail is important for translation efficiency [25]. The 3’ UTRs containing the SV40 polyA sequence may interfere with the formation of the translation initiation complex. This might be due to proteins or microRNAs that bind to the SV40 polyA sequence of the *egfp* transcripts, thereby interfering with the translation initiation for efficient protein synthesis [24,26].

Insect cells use the cleavage/polyadenylation system whereas the SV40 polyA signal provides the necessary signals for cleavage/polyadenylation in mammalian cells. The SV40 polyA sequence contains three elements of AAUAAA for CPSF binding and a downstream GU-rich region for CstF binding as well as a seven-T element for potential transcription termination [27,28]. In an *in vitro* study of baculovirus late gene transcription termination, it was reported that the seven-T region of globin gene transcribed by the viral RNA POL signals the termination process and the end of the globin transcript was terminated downstream of the seven-U motif [28]. When the SV40 polyA signal was inserted downstream of the *egfp* gene but upstream of the putative polyA signal of the *polh* gene, the *egfp* mRNA faithfully formed a 3’end that contains both the polyA signal AAUAAA and the seven-U motif (Fig 5). When the SV40 polyA signal was not inserted, the *egfp* mRNA formed a 3’ end that contains the AAUAAA and terminated at 16 or 22 nucleotides downstream of AAUAAA (Fig 5). One interesting observation in this study is that no RACE product could be detected when the *polh* promoter *egfp* expression cassette was inserted at the *gp37* locus for *egfp* expression whereas at other loci, such as *polh* and *egt*, discrete RACE products were obtained (Fig 3A). The lack of a discrete RACE product from the AcMNPV *gp37* UTR in the Sf21 infection might be due to amplification of multiple 3’ RACE products of different lengths since at least three PCR products were amplified using the forward GFP-486F primer paired with the reverse primers gp37R1, gp37R2 or gp37R3 located immediately upstream of the AAUAAA sequence (Figs 1 and 6C). This result also supports the early report that different lengths of AcMNPV *gp37* mRNA are produced during Sf21 cell infection [29]. The exact reason why no discrete RACE product could be produced from the 3’ UTR of *gp37* is still unknown. However, one possible explanation is that the 3’ processing signals in the *gp37* locus are not as efficient as that of *polh* and *egt* as well as SV40 polyA. For the GU-rich region downstream of the cleavage site, it was reported that a dinucleotide UU following an nt G (GUU) of the precursor mRNA is required for strong binding to the CstF for efficient 3’ processing [30]. A comparison of the three GU-rich regions of the *gp37* UTRs with that of *polh*, *egt* and SV40 polyA all showed the GUU sequences for strong CstF binding (Fig 6A, Table 1). This suggests that the GU-rich regions of *gp37* are not responsible for the inefficiency of 3’ end processing. Therefore, the first AAUAAA motif in the *gp37* UTR might not be efficient for CPSF binding. Since all the *polh*, *egt* and SV40 polyA UTRs have a G nucleotide in the dinucleotide right after the AAUAAA sequence, whereas no G nucleotide was found in the dinucleotide of the *gp37* UTR (Fig 6, Table 1). The lack of a G in the dinucleotide after the three AAUAAA motifs of *gp37* UTRs might be responsible for the inefficiency of 3’ end processing (Table 1). This can be tested by site-directed mutagenesis of the first AAUAAAaa to AAUAAAgg using the transfer vector pAcgp37SV40^-^ as the template (Fig 1, Table 1).

Interestingly, when the SV40 polyA signal was inserted downstream of the *egfp* gene at the *polh*, *egt* and *gp37* loci of the AcMNPV genome, an increase of *egfp* transcript levels was detected compared to the recombinant viruses without SV40 polyA by real-time qPCR. (Fig 3). Real-time qPCR analysis suggests that the SV40 polyA plays certain roles in increasing the *polh* promoter-based *egfp* transcript levels in the BEVS. This result also validates the early idea of inserting the SV40 polyA signal to boost mRNA production [27]. However, the reduction of EGFP yields due to the SV40 polyA signal was unexpected. At the same time, our results are also different from the early report that the SV40 polyA signal at the *p10* locus under the *p10* promoter control reduced mRNA production and protein synthesis [13]. It is still unknown how the SV40 polyA signal helps accumulation of more *egfp* transcripts initiated from the *polh* promoter. It is also unknown if the negative correlation of transcription and translation is promoter-dependent. In conclusion, we recommend that the SV40 polyA be replaced with the *polh* polyA sequence for higher protein production in the BEVS. However, the SV40 polyA insertion will be helpful for other application of the BEVS such as virus-mediated RNAi, which the gene expression end products are RNAs.

## Supporting Information

S1 Fig Quantitative analysis of EGFP yields to test the effects of SV40 polyA on EGFP expression in the baculovirus expression vector system. A pair-wise comparison between viral constructs with and without the SV40 polyA in Sf21 cell infection by fluorescence measurement. Sf21 cells were infected by different viral constructs at an m. o. i. of 1 p.f.u/cell and cells were harvested at 72 h post infection for fluorescence emission measurement in triplicate of three independent cell infections. * indicate significant difference at *P* = 0.05. Error bars denote SD.
(TIF)

S1 Table A list of primers used in PCR to analyze the *egfp* gene transcription (DOC)
