# Supplementary figures and images for: The Influence of SV40 polyA on Gene Expression of Baculovirus Expression Vector Systems
Source: PLoS One. 2015 Dec 14;10(12):e0145019. doi: 10.1371/journal.pone.0145019 (PMC4686012; doi:10.1371/journal.pone.0145019)

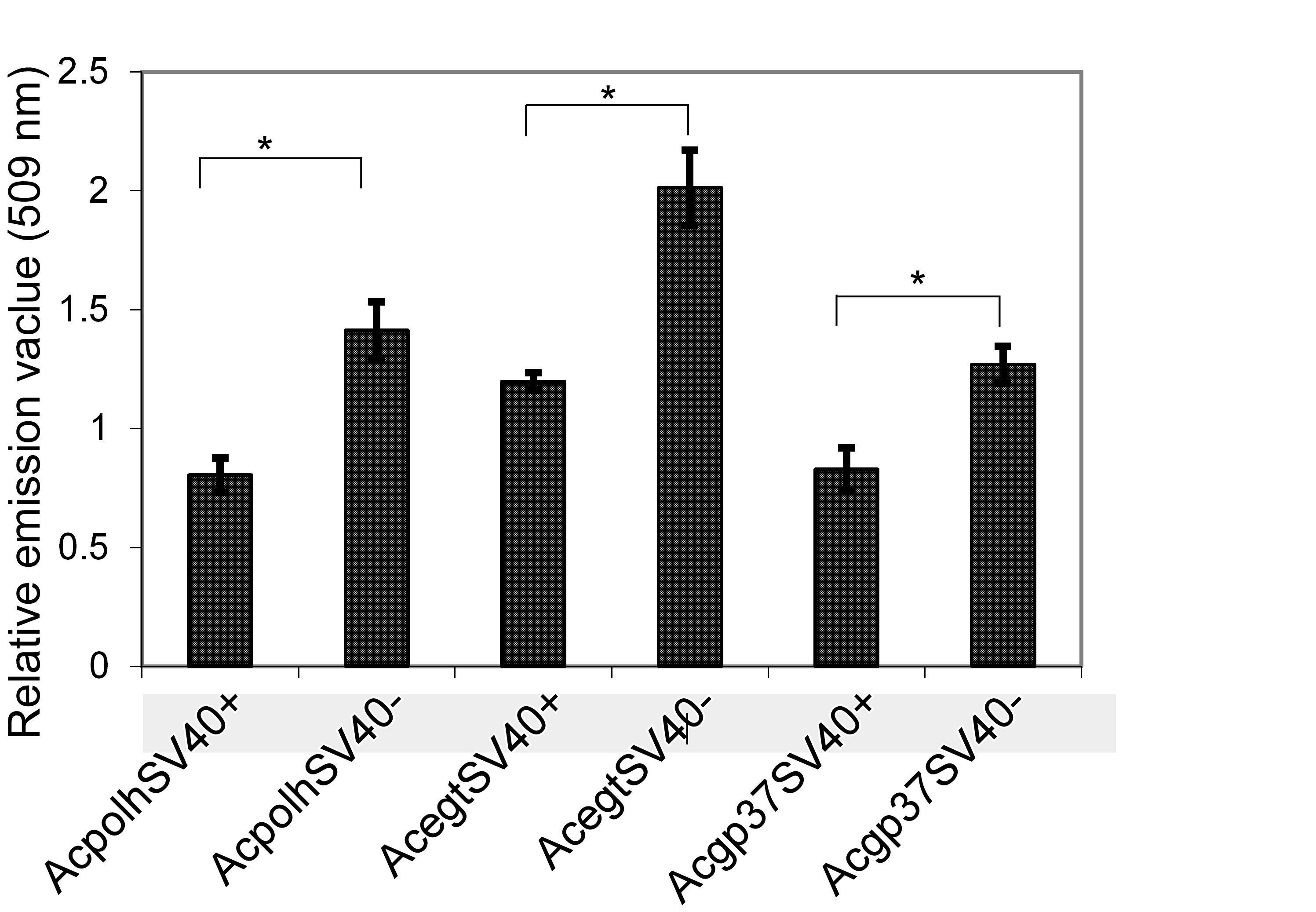

Supplement: S1 Fig — A pair-wise comparison between viral constructs with and without the SV40 polyA in Sf21 cell infection by fluorescence measurement. Sf21 cells were infected by different viral constructs at an m. o. i. of 1 p.f.u/cell and cells were harvested at 72 h post infection for fluorescence emission measurement in triplicate of three independent cell infections. * indicate significant difference at P = 0.05. Error bars denote SD. (TIF) [file pone.0145019.s001.tif]
