# Supplementary material for: The Influence of SV40 polyA on Gene Expression of Baculovirus Expression Vector Systems
Source: PLoS One. 2015 Dec 14;10(12):e0145019. doi: 10.1371/journal.pone.0145019 (PMC4686012; doi:10.1371/journal.pone.0145019)
Supplement: S1 Table — (DOC) [file pone.0145019.s002.doc]

Table S1. A list of primers used in PCR to analyze the *egfp* gene transcription

| Primer names | Primer sequences |
| --- | --- |
| GFP-112F | 5’-GCA AGC TGA CCC TGA AGT TCA TC-3’ |
| GFP-506R | 5’-CGG ATC TTG AAG TTC ACC TTG ATG-3’, |
| SF28S-F | 5’-CGACGTTGCTTTTTGATCCT-3’ |
| SF28S-R | 5’- GCAACGACAAGCCATCAGTA-3’ |
| pGFP-486F | 5’-CAAGGTGAACTTCAAGATCCGCCACAAC-3’ |
| pGFP-639R | 5’-GTTGGGGTCTTTGCTCAGGGCGG-3’. |
| oligo (dT) 3’ RACE adapter primer* | 5'-GAG CAC AGA ATT AAT ACG ACT CAC TAT AGG T12VN-3' |
| 3’ RACE outer reverse primer (outer)* | 5’-GCT GAT GGC GAT GAA TGA ACA CTG-3’ |
| gp37R1 | 5’-GACAGGTGTGAAAATATGGAAG-3’ |
| gp37R2 | 5’-CTATCTTCACTTGAATGGGC-3’ |
| gp37R3 | 5’-CGACGAACGTACAATCATG-3’ |
| gp37R4 | 5’-GACGCAATGGAGGCGTTG-3’ |
| Ac-Polh-F-EcoRI | 5’-GAATTCATGCCGGATTATTCATACC-3’ |
| Ac-Pol-R-XbaI | 5’- TCTAGATTAATACGCCGGACCAG-3 |
| Ac-polhUTR-R-XhoI | 5’-CTCGAGTAACACGCCCGATGTTAAA-3’ |

*Primers from Ambion Inc.
